# Social ecological correlates of workplace sedentary behavior

**DOI:** 10.1186/s12966-017-0576-x

**Published:** 2017-08-31

**Authors:** Sarah L. Mullane, Meynard J.L. Toledo, Sarah A. Rydell, Linda H. Feltes, Brenna Vuong, Noe C. Crespo, Mark A. Pereira, Matthew P. Buman

**Affiliations:** 1 School of Nutrition and Health Promotion, College of Health Solutions, Arizona State University, 425 North 5th Street, Phoenix, AZ 85004 USA; 2 Division of Epidemiology and Community Health, University of Minnesota, 1300 S. 2nd Street, Minneapolis, MN 55454 USA; 3 State of Minnesota Management and Budget, 400 Centennial Office Building, Saint Paul, MN 55155 USA; 4 Fairview Health Services, 2344 Energy Park Drive, Saint Paul, MN 55108 USA; 5 Graduate School of Public Health, San Diego State University, 5500 Campanile Drive, San Diego, CA 92182 USA

**Keywords:** Sedentary behavior, Workplace, Social ecological model, Sector

## Abstract

**Background:**

To identify social ecological correlates of objectively measured workplace sedentary behavior.

**Methods:**

Participants from 24 worksites - across academic, industrial, and government sectors - wore an activPAL-micro accelerometer for 7-days (Jan-Nov 2016). Work time was segmented using daily logs. Sedentary behavior outcomes included time spent sitting, standing, in light intensity physical activity (LPA, stepping cadence <100 steps/min), and in prolonged sitting bouts (>30 min). Outcomes were standardized to an 8 h work day. Two electronic surveys were completed to derive individual (job type and work engagement), cultural (lunch away from the desk, walking at lunch and face-to-face interaction), physical (personal printer and office type) and organizational (sector) factors. Mixed-model analyses with worksite-level clustering were performed to examine multi-level associations. Secondary analyses examined job type and sector as moderators of these associations. All models were adjusted for age, race/ethnicity and gender.

**Results:**

Participants (*N* = 478; 72% female; age: 45.0 ± 11.3 years; 77.8% non-Hispanic white) wore the activPAL-micro for 90.2 ± 15.5% of the reported workday. Walking at lunch was positively associated with LPA (5.0 ± 0.5 min/8 h, *P* < 0.001). Regular face-to-face interaction was negatively associated with prolonged sitting (−11.3 ± 4.8 min/8 h, *P* < 0.05). Individuals in private offices sat more (20.1 ± 9.1 min/8 h, *P* < 0.05), stood less (−21.5 ± 8.8 min/8 h, *P* < 0.05), and engaged in more prolonged sitting (40.9 ± 11.2 min/8 h, *P* < 0.001) than those in public office space. These associations were further modified by job type and sector.

**Conclusions:**

Work-specific individual, cultural, physical and organizational factors are associated with workplace sedentary behavior. Associations vary by job type and sector and should be considered in the design of workplace interventions to reduce sedentary behavior.

**Trial registration:**

Clinical trial No. NCT02566317; ﻿Registered Sept 22nd 2015.

## Background

Sedentary behavior (i.e., waking behavior characterized by an energy expenditure ≤1.5 metabolic equivalents [METs], while in a sitting, reclining or lying posture) [1] is now recognized as a unique health risk factor for cardiometabolic diseases and early mortality [2, 3]. Periods of prolonged sitting without standing or light-intensity physical activity (LPA) acutely and negatively impact circulating blood glucose [4, 5], blood pressure [6, 7] and musculoskeletal pain [8]. Desk-based workers are at particular risk as they spend 70–80% of their workday sitting at a desk [9]. Designing efficacious, feasible, and theory-based workplace sedentary behavior reduction interventions is of public health interest. While a number of studies are using the social ecological framework to reduce occupational sitting [10, 11] we know little regarding the factors at the individual, cultural, physical, and organizational levels that are associated with workplace sedentary behavior and how they may interact.

At the individual level, most research has investigated associations between self-reported sedentary behavior and correlates such as age, race, gender, body mass index (BMI) [12]. However, as suggested by Owen et al., (2011), intervention design is contingent on the sedentary setting [13]. In the context of a workplace setting, preliminary evidence has indicated positive associations between BMI and self-reported workplace sedentary behavior [12, 14]. There is evidence to suggest that workplace sit time may be influenced by job type, with professional/managerial positions (men only) being associated with high levels of workplace sitting [15]. Positive associations between white-collar workers [14, 16], full-time employees [14, 17] and sedentary behavior have also been reported. Recent evidence also suggests that greater work engagement may be associated with less self-reported workplace sitting in men, and to a lesser extent, in women [18]. However, there is a need to examine these workplace specific individual level factors using objective measures of workplace sedentary behavior.

As stated by Owen et al., (2011), the normative climate and worksite culture may influence workplace sedentary behaviors. For example, norms may be implemented socially by questioning why a person may take an active lunch break [13]. Research has indicated that lower levels of perceived job control may be associated with increased occupational sitting [14]. The freedom to take an active lunch break may therefore be representative of worksite culture. Promoting more active lunch breaks (e.g., eating lunch away from the desk, walking at lunch) and encouraging face-to-face interaction have been identified as potential workplace sedentary behavior reduction strategies [19–21]. Each may be influenced by individual preferences and job demands, but also perceived control, management support and organizational structure and may therefore interact in a complex manner across the social ecological spectrum [22]. There is a need to examine these intervention strategies often utilized in workplace interventions and the potential bi-directional influences that may exist at the individual and/or organizational level.

At the physical level, there has been a rapid proliferation of studies examining the impact of ‘activity-permissive’ workstations (e.g., treadmill desks, sit-stand workstations) with the majority published in the last 10 years [23–25]. Differential associations between self-reported sitting break frequency [26] and activity levels [25, 27, 28] according to spatial configuration have been reported. Although insightful, findings are limited by self-report measures of sedentary behavior [26], sample size [25], or lack of diversity in organizational sector [19, 20].

**Fig. 1:**
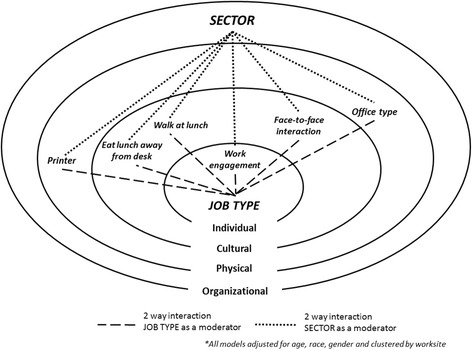
A social ecological, hypothesis-generating framework for workplace sedentary behavior articulating interactional associations across individual, cultural, physical and organizational levels

Using the social ecological model as a hypothesis generating framework (see Fig. 1), our primary objective was to build upon existing research using self-report measures of sitting time, and examine whether workplace specific individual, cultural, physical, or organizational factors were associated with objectively measured workplace sedentary behavior. To better inform future workplace sedentary behavior reduction interventions, our secondary objective was to examine whether job type and/or sector moderated any patterns of associations with workplace sedentary behavior. Although we present hypothesis generating research, we hypothesize that levels of sedentary behavior will be influenced by all levels of the social ecological model and that these associations will differ according to the type of work being done (job type) and the organization within which it is being done (sector).

## Methods

### Study design and procedures

We report cross-sectional associations drawn from baseline data collected January–November 2016, from the Stand & Move at Work cluster-randomized trial (NCT02566317). A full description of the study methods are reported elsewhere [29] and briefly described here. Main worksite eligibility criteria included; a) 20–50 employees with >80% of employees working full time (30+ hours/week); (b) highly sedentary occupations; (c) <10% of employees currently using a sit-stand workstation; (d) leadership willing to be randomized; and (d) able to have sit-stand workstations installed. Following enrollment of the worksite, all employees within the worksite were invited to be screened for eligibility via a self-administered questionnaire. Additional eligibility criteria included (a) 18 years or older; (b) generally good health and able to safely reduce sitting and increase LPA; (c) not currently pregnant. Participants were recruited from twenty-four worksites across academic, industry and government sectors (eight worksites per sector) - in the greater Minneapolis/St. Paul and Phoenix metropolitan regions. Study procedures were approved by the institutional review boards of Arizona State University and the University of Minnesota, and informed consent was provided by all worksites and employees prior to participation.

### Social ecological factors

Demographic data, job category and printer prevalence were derived from an online survey via Qualtrics (Salt Lake City, UT) and completed during the baseline assessment week. While eight job categories were listed in the demographic survey, three were not applicable to our sedentary, office-based population due to both worksite and participant eligibility criteria (i.e. service occupations, operators and laborers). Of the remaining five categories, the clerical, technical support, and sales options were collapsed into a single ‘*clerical’* category. Participants were categorized into one of three job types: executive, professional or clerical. This is comparable to a recent cluster randomized trial in which job categories were defined as manager/administrator, professional/associate and clerical/sales/service [20].

The Utrecht Work Engagement Survey (short form UWES-9) was used to assess levels of work engagement via nine questions on a 7-point Likert-type scale (0–6) targeting three constructs: vigor, absorption and dedication [30]. High correlations and internal consistencies [30, 31] have been reported between all 9-items, thus, as an accepted measure of work engagement when using the shortened version [30], the mean UWES score was calculated for each participant. A higher UWES score represented a higher level of work engagement. The frequency at which participants engaged in workplace behaviors like eating lunch away from their desk, walking at lunch, and face-to-face interactions, were assessed using 5-point Likert-type scales (*Never* [1] to *Always* [5]). These questions were previously used in a cluster randomized trial to better characterize the workplace [32]. An additional online survey was completed by participants to derive whether participants were located in a public (e.g., cubicle, open space) or private office (e.g., enclosed, not shared).

### Sedentary behavior in the workplace

Participants wore an activPAL-micro accelerometer (PAL Technologies, Glasgow, United Kingdom) continuously for seven days. The activPAL provides a valid and reliable measure of posture (sitting vs. standing) for free-living settings [33]. Work time was identified and extracted using work logs which were administered daily by email. Workplace sedentary behavior outcomes were sit time, stand time, light-intensity physical activity (LPA, stepping <3.0 metabolic equivalents), and time in bouts of prolonged sitting (>30 min). All outcomes were standardized to an 8 h work day.

#### Data analyses

Statistical analyses were completed using SAS Version 7.1, SAS Institute, Inc., (Cary, North Carolina). The activPAL data were assessed for normality, linearity and heteroscedasticity. Models showed no evidence of collinearity (i.e., variance inflation factor < 2), non-linearity, non-normality, or heteroscedasticity as assessed by scatterplots. Between sector differences were examined via chi-square tests for categorical variables and one-way analysis of variance (ANOVA) for continuous variables. To model complex multi-level associations between individual factors, multi-level models (SAS Procedure ‘PROC MIXED’) were fitted and clustered by worksite to assess each dependent variable (including the number of minutes spent sitting, standing, in LPA, in bouts of prolonged sitting [>30 min]). The first model (base model) included all factors listed in Fig. 1 as main effects. The second model included all two-way interactions to examine both job type and sector as moderators of the associations found in the base model. The third model included three-way interactions via a backward elimination process (removing two-way interactions from the model if their *p*-value was >0.05) to examine three way associations between job type, sector and remaining factors. The median split was used to dichotomize work engagement into high and low engagement for ease of interpretation. All models were adjusted for age, race/ethnicity and gender.

## Results

### Descriptive results

At the cluster level, 56 worksites were invited to participate, 11 were not interested at the time, 21 were ineligible and 24 were enrolled. At the individual level, 1312 employees were invited to participate, 394 people did not respond, 906 completed the eligibility survey, 756 were eligible and 641 participants were enrolled in the Stand & Move at Work study. For these cross-sectional baseline analyses, only participants who completed both the environmental and demographic surveys and had valid activPAL data were included (*N* = 478). Table 1 shows the demographics, individual, cultural, physical factors and sedentary behaviors stratified by sector. The sample population was predominantly female, non-Hispanic white, and reported mostly professional job types. Chi-square analyses and one-way ANOVA pairwise comparisons indicated that participants were distributed relatively equally across worksite sectors, with no significant differences detected in age, race/ethnicity, gender, and job type. Significant workplace sector differences in the assessed factors and behaviors are highlighted in Table 1.

**Table 1:** Description of individual, cultural, physical factors, sedentary behaviors and between sector comparisons

	Academic ^a^	Industry ^a^	Government ^a^	Total
**Descriptives**				
N	171 (35.8)	139 (29.1)	168 (35.2)	478
Age	44.5 [11.3]	45.3 [11.4]	45.2 [11.3]	45.0 [11.3]
Race				
White Non-Hispanic	135 (28.2)	115 (24.1)	122 (25.5)	372 (77.8)
White Hispanic	7 (1.5)	6 (1.3)	22 (4.6)	35 (7.3)
Black	9 (1.9)	6 (1.3)	9 (1.9)	24 (5.0)
Asian	12 (2.5)	7 (1.5)	9 (1.9)	28 (5.9)
Other	8 (1.7)	5 (1.1)	6 (1.3)	19 (4.0)
Gender				
Male	54 (11.3)	31 (6.5)	47 (9.8)	132 (27.6)
Female	117 (24.5)	108 (22.6)	121 (25.3)	346 (72.4)
**Individual-level factors**				
Job type				
Executive	22 (4.6)	23 (4.8)	22 (4.6)	67 (14.0)
Professional	91 (19.0)	76 (15.9)	105 (22.0)	272 (56.9)
Clerical	58 (12.1)	40 (8.4)	41 (8.6)	139 (29.1)
Work engagement	4.1 [1.1]*	4.1 [0.9]	4.4 [0.9]	4.2 [1.0]
**Psychosocial behaviors**				
Walking at lunch	1.6 [1.2]	1.4 [1.0]*	1.8 [1.3]	1.6 [1.2]
Lunch away from desk	1.9 [1.3]	1.8 [1.3]	2.1 [1.2]	1.9 [1.3]
Face-to-face interaction	2.4 [0.8]	2.2 [0.9]*	2.6 [0.7]	2.4 [0.8]
**Micro-environmental factors**				
No printer	127 (26.6)	111 (23.2)	154 (32.2)	392 (82.0)
Printer	44 (9.2)	28 (5.9)	14 (2.9)**	86 (18.0)
Public office	102 (21.3)	104 (21.8)	124 (25.9)	330 (69.0)
Private office	69 (14.4)	35 (7.3)**	44 (9.2)	148 (31.0)
**Sedentary behaviors** ^**b**^				
Sitting	328.0 [72.1]	344.4 [88.4]	325.8 [78.7]	332.0 [79.6]
Standing	114.4 [70.0]	106.0 [85.5]	111.9 [75.3]	111.1 [76.5]
LPA	31.4 [14.2]*	24.7 [11.8]*	35.7 [16.4]	31.0 [15.0]
Sitting >30 min	139.2 [84.3]	164.5 [102.2]***	148.4 [82.6]	149.8 [89.7]

Boldface indicates statistical significance for chi-squared and one-way ANOVA tests (*P* < 0.05); Bonferroni analyses

^a^ values are either N (%) or mean ± [SD]

^b^ Sedentary behavior variables are expressed as minutes per 8 h workday

* Significantly lower than government sector

** Significantly lower than academic sector

*** Significantly higher than academic sector

### Correlates of workplace sedentary behavior (main effects)

**Fig. 2:**
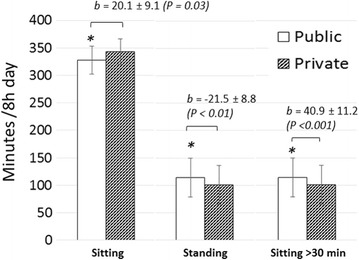
Predicted mean levels of sitting, standing and prolonged sitting per 8 h work day according to office type

Mixed-model results are presented in Table 2. In the base model, no significant main effects were detected for individual level factors such as job type or work engagement. At the cultural level, significant main effects were detected for walking at lunch and face-to-face interaction. A higher frequency of walking at lunch was associated with increased levels of LPA (5.0 ± 0.5 min/8 h, *P* < 0.001) and less prolonged sitting (−7.8 ± 3.6 min/8 h, *P* = 0.025). Higher levels of face-to-face interaction were associated with less time in bouts of prolonged sitting (−11.3 ± 4.8 min/8 h, *P* = 0.015). At the physical level, significant main effects were detected for office type across several outcomes (see Fig. 2). Private offices were associated with more sitting (20.1 ± 9.1 min/8 h, *P* = 0.013), less standing (−21.5 ± 8.8 min/8 h, *P* = 0.012) and more prolonged sitting (40.9 ± 11.2 min/8 h, *P* < 0.001). A significant main effect was also detected for sector, in which more sitting was observed in industry compared to the academic sector (20.3 ± 9.2 min/8 h, *P* = 0.044).

**Table 2:** Social ecological factors and their associations with workplace sedentary behavior ^a^

	Sitting time b [95% Cl]	Standing time b [95% Cl]	LPA b [95% Cl]	Sitting >30 min b [95% Cl]
Base model				
**Individual factors**				
Job type				
Executive (ref)				
Professional	2.9 [−18.9, 27.5]	−3.8 [−27.3, 17.7]	1.1 [−2.8,4.6]	−5.2 [−26.7, 24.2]
Clerical	9.2 [−19.2, 34.4]	−8.4 [−33.2, 18.7]	0.4 [−3.7, 5.1]	−3.5 [−34.1,25.7]
Work engagement	−4.0 [−9.9, 5.1]	3.6 [−5.4,9.1]	−0.1 [−1.3, 1.1]	−4.2 [−10.2, 6.2]
**Cultural factors**				
Lunch away from desk	−2.9 [−8.1. 3.7]	2.0 [−4.1, 7.4]	0.5 [−0.3, 1.6]	−3.0 [−8.3,4 6]
Walking at lunch	−2.5 [−8 8,3.7]	−3.1 [−9.4,27]	5.0 [4.0, 6.0]***	−7.8 [−14.5, −0.8]*
Face to face interaction	−3.8 [−15.2,2.2]	2.3 [−3.6,13.3]	0.5 [−0.8, 1.9]	−11.3 [−21.0, −2.3]*
**Physical factors**				
No Printer (ref)				
Printer	0.6 [−14.0, 26.3]	−1.7 [−26.1, 12.8]	1.8 [−1.5, 5.5]	−6.9 [−23.6, 23.7]
Public office (ref)				
Private office	20.1 [4.9,41.0]*	−21.5 [−39.8, −5.1]*	1.6 [−2.2,4 2]	40.9 [19.7,62.6]***
**Organizational factors**				
Academic (ref)				
Industry	20.3 [0.4,39.2]*	−15.2 [−32.1,4.7]	−5.9 [−12.7, 0.0]	25.2 [−7.0, 61.9]
Government	14.2 [−9.6, 28.1]	−17.1 [−29.8, 6.0]	3.5 [−3.0, 9.7]	21.3 [−13.6, 54.6]
Two-way interactions				
*Moderator: Job type*				
Professional x Printer	73.3 [14.0,130.2]*	−68.8 [−123.8, −10.7]*		
*Moderator: Sector*				
Government x Lunch away from desk			−2.7 [−5.0, −0.5]*	
Government x Walking at lunch			3.7 [1.6,6.1]**	
Government x Private office	51.9 [14.3, 93.6]**	−44.6 [−85.3, −8.2]*	−6.9 [−13.5, −0.2]*	
3 -wav interactions				
*Moderators: Sector < & Job type*				
Industry x Clerical x Work engagement				38.5 [1.3, 77.9]*
Industry x Clerical x Private office			−24.2 [−48.3, −0.6]*	

All models are adjusted for age, race ethnicity, and gender; LPA = Light-intensitv physical activity; only significant associations are presented for two- and three-way interactions

^a^ Sedentary behavior variables are expressed as minutes per 8 h workday. Boldface indicates statistical significance

*P < 0.05

**P < 0.01

***p < 0.001

### Job type and sector as moderators of the social ecological – Sedentary behavior associations (2-way interactions)

All significant 2-way interactions are presented in Table 2. In general, the patterns of association between sedentary behaviors and factors spanning the social ecological model were similar across job categories. However, a significant interaction was detected for professional employees with personal printers, whereby they exhibited more sitting (73.3 ± 29.4 min/8 h, *P* = 0.015) and less standing (−68.8 ± 28.5 min/8 h, *P* = 0.020), relative to executive employees with personal printers. Sector emerged as a moderator of several factors and outcomes. At the cultural level, eating lunch away from the desk was associated with less LPA in the government sector when compared to the academic sector (−2.7 ± 1.1 min/8 h, *P* = 0.018). In contrast, the pattern of association between increased LPA and walking at lunch, was more pronounced in the government sector (3.7 ± 1.1 min/8 h, *P* = 0.009). At the physical level, levels of sitting were markedly higher (51.9 ± 20.0 min/8 h, *P* = 0.008), and levels of standing (−44.6 ± 20.0 min/8 h, *P* = 0.018) and LPA (−6.9 ± 3.4 min/8 h, *P* = 0.045) markedly lower, for government employees in private offices, when compared to academic employees in private offices.

**Fig. 3:**
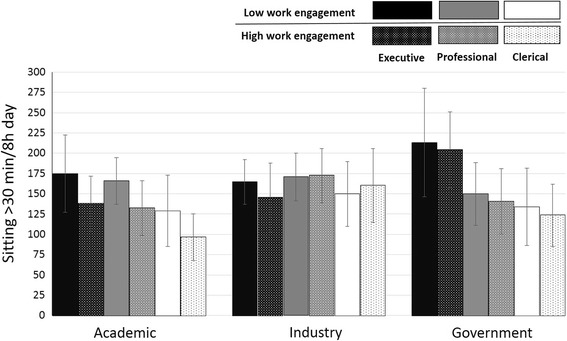
Median split derivation of high and low work engagement levels to illustrate three- way interaction between mean work engagement, job type and sector

All significant 3-way interaction results are presented in Table 2. At the individual level, the exploratory results indicated a significantly different pattern of association between levels of work engagement and prolonged sitting for clerical employees in the industry sector. High levels of work engagement were associated with less prolonged sitting in both the academic and government sector, across all job types (see Fig. 3). The opposite pattern of association was detected for clerical employees in industry, in which high levels of work engagement were associated with increased bouts of prolonged sitting (38.5 ± 19.4 min/8 h, *P* = 0.043). Differential associations between office type and levels of LPA were also detected for clerical employees in industry in which the negative association between private offices and LPA was more pronounced for clerical employees in industry when compared to other job types and sectors (−24.2 ± 12.0 min/8 h, *P* = 0.045).

## Discussion

Although exploratory in nature, the analyses elicited insightful results. Our results support cross-sectional analyses conducted by Duncan et al., (2015) who concluded that workplace sedentary behaviors may be differentially influenced by multi-level social ecological factors [26]. Firstly, the base model results indicated that eating lunch away from the desk may have less impact on sedentary behaviors than walking at lunch, which was positively associated with increased LPA. Although the behaviors may align temporally, they were not highly correlated (*r* < 0.4) and are likely driven by different motivations. These results contrast null findings reported for policy level workplace interventions specifically targeting walking groups and walking meetings [34, 35]. The non-significant decreases in mean sitting time reported may be attributed to the fact that their outcomes focused on sitting only, not LPA. Walking at lunch may be considered a workplace strategy to improve levels of LPA rather than sit-time alone.

Level of face-to-face interaction was negatively associated with prolonged bouts of sitting. Our cross-sectional analyses contrast the null findings reported in a multi-component intervention which compared sit-stand desk use in an intervention versus control group [19]. The application of this strategy likely requires organizational support and potential policy level change, and it is not clear how face-to-face interaction was specifically encouraged within this intervention beyond the addition of a sit-stand desk. More recently, several cluster randomized trials have targeted face-to-face interaction within larger multi-component interventions [20, 21], but this strategy has not been clearly isolated as a predictor of workplace sedentary behavior. Our findings suggest that more detailed methods of face-to-face interaction assessment and evaluation may be warranted in future workplace intervention research.

The most consistent association for workplace sedentary behavior was observed at the physical level for office type. Private offices, compared to public, were associated with more sitting, more prolonged sitting, and less standing. These results were independent of job type and partially support previous findings for sitting break frequency, in which it was concluded that correlates of sedentary behavior differed by office type [26]. *Reciprocal Determinism* [36], one of the key constructs of Social Cognitive Theory in which a person may be both an agent for, and responder to, change, may be more prevalent in open plan offices and such social cues may be less prevalent in private offices. It should therefore be considered that those in private offices may not receive the full ‘dose’ of multi-component workplace interventions. This is further supported by research examining office spatial design which indicated high correlation between co-presence and visibility of fellow workers as a visual ‘cue’ to encourage face-to-face interaction [27]. Although our 2-way and 3-way interaction analyses did not indicate significant associations between office type and the level of face-to-face interaction, office type (whether public or private) should be considered when determining strategies for increasing interaction among employees.

The 2-way interaction results for job type suggest that the purpose of a personal printer may be more influential than the presence of a printer alone. Researchers should aim to collect more contextual data to determine how often, and for what purpose the printer is used (personal use vs. distributing documents to others). The need for contextual data was also reinforced when examining sector as a moderator, which revealed conflicting results. Eating lunch away from the desk was associated with less LPA for government employees, however, walking at lunch was associated with markedly higher LPA. Collecting additional contextual data, such as; where lunch is eaten when not eaten at the desk, how far away the location is, and how long the lunch break is (strict policy vs. none) would provide a better indicator of cultural influences on workplace sedentary behavior across sectors, particularly as meal break laws vary by both state and employer [37]. Moreover, associations between neighborhood walkability and reduced sedentary behavior have been reported [38], this suggests that the built environment surrounding the worksite (which may not be conducive to walking) may also influence lunch break behaviors [39]. However, we did not assess this metric and posit that researchers should assess the ‘walkability’ of the surrounding workplace environment for future workplace intervention analyses [40].

A significant interaction effect was detected for sector and private offices which indicated that the positive associations between sedentary behavior and private offices were driven by the government sector. The existence of specific policies regarding workspace utilization and allocation according to job type in the government sector in which executives and directors are allocated private offices [41], suggests that the workplace micro-environment may be highly representative of both job type and management level and therefore a stronger moderator of sedentary behavior in the government sector.

Our 3-way interaction findings indicated that there were changes in the associations between levels of work engagement and job type across sectors. Higher levels of work engagement were associated with less prolonged sitting in academic and government employees, which partially supports previous findings [18]. However, the opposite pattern of association was observed for clerical employees in industry. Possible inferences may be that tasks may be considered more time-sensitive within industry, organizations may be more hierarchal in nature, and the perception that ‘being at your desk means higher engagement’ may be more prevalent.

Our interaction results align with acknowledged limitations of recently completed cluster randomized trials, which although represent the most definitive research to date, do not explore potential differential relationships or tackle the heterogeneity of workplaces [20, 21]. Specifically, Healy et al., (2016) [20] acknowledge limited generalizability due to recruitment within one single organization. Similarly, although Danquah et al., (2016) [21] recruited across public and private sectors, stratification or moderator analyses were not conducted by sector. Further, it was reported in the Cochrane Review 2016 [42], that a ‘sedentary workforce’ has been well represented in the research conducted to date, but work environments, cultures and ‘norms’ vary greatly, and the acceptability and feasibility of workplace interventions pertaining to sedentary behaviors may differ accordingly. Our exploratory analyses support this conclusion and suggest that bi-directional associations may exist between individual, cultural, physical and organizational factors.

### Strength and limitations

This study has several strengths. Firstly, our large sample size with objectively measured levels of sedentary behavior builds upon the research conducted to date which has been limited by either sample size [25] or self-report measures of sedentary behavior [26]. Secondly, our mixed-model analyses allowed us to explore interactions and complex associations across multiple levels of the social ecological model. Finally, worksites were also recruited equally across three different sectors, allowing for the examination of these organizational-level influences. The main limitation of our research is that our observations cannot be viewed as directional or causal due to the cross-sectional and exploratory nature of this research. We acknowledge that we can only hypothesize explanations for patterns of associations. We further acknowledge that worksite culture is a highly influential social ecological level that could not be well explored within our research due to lack of supporting contextual data. Our methods did not allow us to parse out the ‘level of perceived control’ which may be more indicative of worksite culture and/or organizational policies [14]. Another limitation was the reduction in our sample size from 641 to 478 due to poor micro-environment survey completion; combining the surveys to reduce participant burden may have achieved a higher completion rate. Finally, although we recruited across three sectors, our sample comprised of predominantly white, non-Hispanic females, with highly sedentary office based jobs, which restricts the generalizability of our results. Nonetheless, our findings generate important hypotheses examining objectively measured levels of sedentary behavior, to be tested in future experimental and longitudinal research.

## Conclusions

It is understood that health behaviors are shaped through a complex interplay of determinants at various levels and social ecological models suggest that these multiple levels of influence interact across levels. Our hypothesis generating approach aimed to further investigate the bi-directional associations that may span the social ecological spectrum and influence levels of sedentary behavior in the workplace. At the simplest level, a ‘sedentary workforce’ may be considered a homogenous population, with all employees meeting a set criteria of sedentary time during a typical work day. However, our findings suggest that both between, and within, workplace variation may exist at all levels of the social ecological spectrum. Future behavioral studies across diverse job types and sectors should be encouraged. Although exploratory in nature, such analyses are needed to identify marginal relationships to both inform and evaluate intervention design if they are to be of high ecological validity. We should endeavor to diversify recruitment and examine existing factors across the social ecological spectrum in order to maximize the effectiveness of workplace sedentary behavior reduction strategies via more tailored processes.

## Abbreviations

ANOVA: Analysis of Variance
LPA: Light-intensity Physical Activity
METs: Metabolic Equivalent of Task(s)
UWES: Utrecht Work Engagement Survey
